# Plasma proteomics detects hyperlipoprotein A in a patient with recurrent lipomas

**DOI:** 10.1016/j.jdcr.2025.12.013

**Published:** 2025-12-15

**Authors:** Baruch Kaplan, Andrea Volk, Wendee Duong, Meshora Suthanthira, Sabrina Khalil, Jack L. Arbiser

**Affiliations:** a Ariel University School of Medicine, Metroderm/United Dermatology Partners, Atlanta, Georgia; b Philadelphia College of Osteopathic Medicine, Suwanne, Georgia

**Keywords:** apoliporptein A, lipids, lipoma, proteomics, recurrence

## Introduction

Lipomas are exceedingly common, but the pathogenesis of lipomas is not fully understood. Genetic syndromes account for a small minority of lipomas, and somatic mutations are likely to account for the majority of lipomas. While biologically benign, lipomas can be disfiguring and/or painful. Surgical excision is the therapy of choice for lipomas, and recurrence is uncommon. We present a case of a patient with rapid recurrence of lipomas after multiple attempts at excision. Given that this lesion was histologically benign, we hypothesized that a circulating factor might be responsible for this unusual behavior of the lipoma. Proteomics was performed using plasma from the patient and her husband as a normal control. Proteomics demonstrated that the patient had elevated levels of apolipoprotein A (15 fold) compared with her husband. We demonstrate the utility of proteomics in elucidating circulating factors which may play a role in dermatologic conditions. Second, hyperlipoproteinemia A may be a cardiac risk factor. Finally, patients with lipomas may benefit from testing and subsequent cardiology follow up.

## Case history

**Fig 1:**
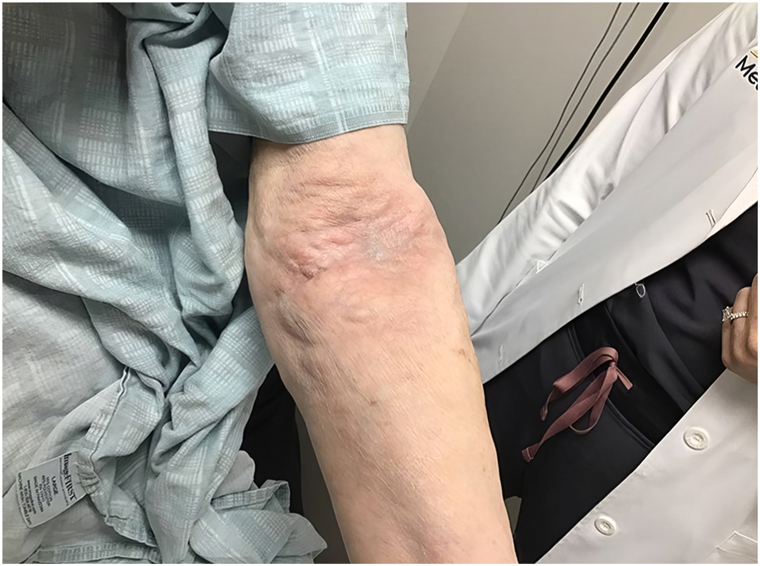
Clinical presentation of lipomas in the popliteal fossa.

**Fig 2:**
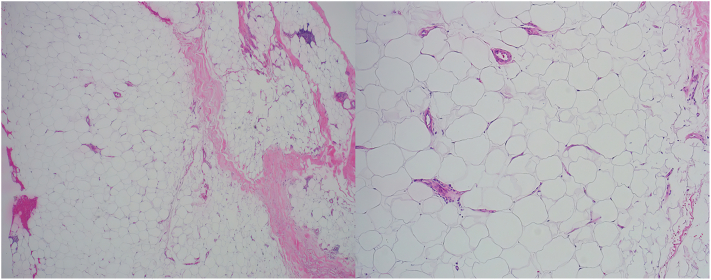
Lipoma (low magnification, right, high magnification, left). Well-circumscribed lesion composed of uniform mature adipocytes arranged in lobules, separated by thin fibrous septa (H&E stain, low magnification). (*Right*) Uniform mature adipocytes with clear cytoplasm and eccentrically located nuclei, without atypia or mitotic activity, consistent with benign lipoma (H&E stain, high magnification).

The patient is a 71 year old female who presented with a 2 month history of thickening of the skin in her left antecubital fossa (Fig 1). Her past medical history is notable for arthritis, cerebrovascular accident, hypertension, hypercholesterolemia, and hypothyroidism. Medications include amlodipine, hydrochlorothiazide, levothyroxine, and losartan. Differential diagnosis included fibrotic processes and mucinosis. Serum protein electrophoresis was obtained and showed no abnormality. Punch biopsy was obtained, revealing mature adipocytes, consistent with a lipoma (Fig 2). Four attempts were made to excise the lipoma, each followed by rapid regrowth. After these attempts, the patient elected not to attempt further surgical removal, and the lesion remained stable in size. Blood was drawn from the patient and her husband, spun for plasma, and submitted to the Emory Integrated Proteomics Core for nanocapillary liquid chromatography coupled with tandem mass spectrometry. The patient denied of any history of local steroid injection and HIV.

## Discussion

Cutaneous lipomas are exceedingly common neoplasm of the skin.^1^ While most lipomas do not need treatment, lipomas are excised for concerns of pain and appearance. Excision is curative in >99% of cases, and even when lipomas are incompletely removed, regrowth is uncommon. The case we describe is novel because of rapid regrowth of lesions within weeks of excision, and a histologically benign appearance. We thus hypothesized that a circulating factor may be playing a role in the regrowth in this patient. Little is understood about the factors responsible for lipoma growth. In order to determine potential growth factors for lipomas, we used comparative proteomics, comparing factors between the patient and her husband as a negative control. While there are obvious differences in using serum of opposite genders as a control, they share a common age, microbiome, and potential diet. We demonstrate the utility of proteomics to provide a possible mechanistic explanation for uncommon dermatologic findings, and this may be generally applicable to finding circulating factors in other unexplained dermatologic disorders.

We ranked the top 15 disparate circulating factors between the patient and her spouse (Table I). Of note, apolipoprotein (a) shows the highest difference in expression, with a 15 fold elevation in the patient versus her spouse. Apolipoprotein (a) has been implicated in lipomagenesis as well as being a cardiac risk factor. Musaad et al observed hyperlipoproteinemia in a large paraspinal lipoma, and levels of apolipoprotein (a) did not decrease after resection, suggesting that the lipoma itself is not the source of apolipoprotein (a).^2^ Elevated levels of apolipoprotein (a) have also been observed in multiple symmetric lipomatosis.^3^^,^^4^

**Table I:** Comparison of plasma peptides between patient and spouse, with ratio of expression at right

Accession	Protein name	Patient	Spouse	Ratio
P08519	Apolipoprotein (a)	30	2	15.0
P35443	Thrombospondin-4	9	2	4.5
P01780	Immunoglobulin heavy variable 3-7	4	1	4.0
Q13201	Multimerin-1	4	1	4.0
P55058	Phospholipid transfer protein	7	2	3.5
A0A0B4J1X5	Immunoglobulin heavy variable 3-74	3	1	3.0
P22105	Tenascin-X	6	2	3.0
P18428	Lipopolysaccharide-binding protein	8	3	2.7
Q6UXB8	Peptidase inhibitor 16	8	3	2.7
P19320	Vascular cell adhesion protein 1	13	5	2.6
Q9Y5Y7	Lymphatic vessel endothelial hyaluronic acid receptor	5	2	2.5
P33151	Cadherin-5	7	3	2.3
O14791	Apolipoprotein L1	6	3	2.0
P01034	Cystatin-C	14	7	2.0
P49747	Cartilage oligomeric matrix protein	4	2	2.0

Apolipoprotein (a) is regarded as “good” lipids, as it is part of high-density lipoproteins. However, our patient had a history of significant cardiovascular disease, suggesting that it was not protective for her. There is an emerging consensus that extremely high levels of apolipoprotein (a) may be deleterious^5^^,^^6^ and patients with large or multiple lipomas should be screened for high levels of apolipoprotein (a) and subsequent cardiology surveillance. Extensive work from the Gelfand laboratory has shown that psoriasis is a cardiac risk factor, and our work suggests that lipomas may be an additional cutaneous finding to warrant cardiology surveillance.^7^ The main limitation of this study is the use of a single control, the patient’s spouse. Although this choice decreased variability in age, microbiome, and potential diet, sex-specific, and individual biological differences may influence circulating protein levels. The observed proteomic differences could reflect individual variation rather than disease-specific mechanisms. While a 15-fold elevation in apolipoprotein (1) appears striking and suggests potential biological relevance, it should be interpreted cautiously and confirmed in a larger cohort with age- and sex-matched controls. Additionally, we acknowledge that local factors cannot be excluded. Small residual lipoma tissue may persist and secrete growth-promoting signals and contribute to recurrence.^8^ Therefore, circulating and local mechanisms are both plausible and may act synergistically. Our findings suggest a two-hit phenomenon, in which a lipoma develops due to a somatic mutation, and then enlarges due to high levels of circulating apolipoprotein (a). Finally, proteomics offers a rapid method of assessing the role of circulating factors in systemic dermatologic disease and could be used for assessing responses to systemic therapies.

## Conflicts of interest

None disclosed.
